# Innate Immune Responses by Respiratory Viruses, Including Rhinovirus, During Asthma Exacerbation

**DOI:** 10.3389/fimmu.2022.865973

**Published:** 2022-06-20

**Authors:** Kazuyuki Nakagome, Makoto Nagata

**Affiliations:** ^1^Department of Respiratory Medicine, Saitama Medical University, Saitama, Japan; ^2^Allergy Center, Saitama Medical University, Saitama, Japan

**Keywords:** bronchial asthma, eosinophils, epithelial cell-related cytokines, innate lymphoid cells, rhinovirus

## Abstract

Viral infection, especially with rhinovirus (RV), is a major cause of asthma exacerbation. The production of anti-viral cytokines such as interferon (IFN)-β and IFN-α from epithelial cells or dendritic cells is lower in patients with asthma or those with high IgE, which can contribute to viral-induced exacerbated disease in these patients. As for virus-related factors, RV species C (RV-C) induces more exacerbated disease than other RVs, including RV-B. Neutrophils activated by viral infection can induce eosinophilic airway inflammation through different mechanisms. Furthermore, virus-induced or virus-related proteins can directly activate eosinophils. For example, CXCL10, which is upregulated during viral infection, activates eosinophils *in vitro*. The role of innate immune responses, especially type-2 innate lymphoid cells (ILC2) and epithelial cell-related cytokines including IL-33, IL-25, and thymic stromal lymphopoietin (TSLP), in the development of viral-induced airway inflammation has recently been established. For example, RV infection induces the expression of IL-33 or IL-25, or increases the ratio of ILC2 in the asthmatic airway, which is correlated with the severity of exacerbation. A mouse model has further demonstrated that virus-induced mucous metaplasia and ILC2 expansion are suppressed by antagonizing or deleting IL-33, IL-25, or TSLP. For treatment, IFNs including IFN-β suppress not only viral replication but also ILC2 activation *in vitro*. Agonists of toll-like receptor (TLR) 3 or 7 can induce IFNs, which can then suppress viral replication and ILC2 activation. Therefore, if delivered in the airway, IFNs or TLR agonists could become innovative treatments for virus-induced asthma exacerbation.

## Introduction

Viral infection is extensively involved in the exacerbation of asthma (1, 2). Viral infection is identified in 50–80% of patients with asthma exacerbation, and rhinovirus (RV) is detected in 50–80% of patients in whom the causative virus has been identified (3). Bronchial asthma is a chronic disease characterized by airway hyperresponsiveness (AHR), a variable degree of airway obstruction, and eosinophilic airway inflammation (4, 5). Although various types of cells are involved in this process, both neutrophil and eosinophil inflammation may contribute to the development of viral-induced asthma exacerbation.

Innate immune responses contribute to the pathogenesis of eosinophilic airway inflammation. Type 2 innate lymphoid cells (ILC2) activated by epithelial cell-related cytokines such as IL-33, IL-25, and thymic stromal lymphopoietin (TSLP) (6, 7) can induce IL-5 and IL-13 and thus eosinophilic inflammation. This indicates that innate immune responses including ILC2 may play important roles in virus-induced airway inflammation and asthma exacerbation.

In the present review, the role of viral infection and innate immune responses, including the role of ILC2, in asthma exacerbation is discussed.

## Role of Viral Infection in the Development of Asthma Exacerbation

Viral infection, especially RV infection, plays an important role in the pathogenesis of asthma exacerbation. RVs have tremendous diversity, and there are about 100 classical serotypes that are classified into RV species A (RV-A) and RV-B (1, 2). With the development of molecular biological technologies such as PCR, more than 60 new RVs have been discovered (almost all are RV-C) (1, 2). RV viral capsid includes VP1, VP2, VP3 and VP4 proteins, and VP1 and VP3 are important for attachement to cell surface receptors. The receptor for the major group of RV-A and for all of RV-B is intercellular adhesion molecule (ICAM) 1, while that for the minor group of RV-A is low-density lipoprotein receptor, and that for RV-C is cadherin-related family member 3 (CDHR3) (8, 9) (**Table 1**). RV is taken up by receptor-mediated endocytosis and replicates in airway epithelial cells. Generally, the components of the viruses are recognized by pattern recognition receptors (PRRs) such as toll-like receptors (TLRs), melanoma differentiation-associated protein (MDA) 5, and retinoic acid-inducible gene (RIG) 1-like receptors. RV is a positive-sense single-strand (ss) RNA virus, however, it could be double-strand (ds) RNA during replication process. Therefore, dsRNA is recognized by TLR3 and ssRNA is recognized by TLR7/8 in endosomes of epithelial cells, which activate myeloid differentiation primary response 88 (MYD88) or TIR-domein-containing-adaptor-inducing interferon-β (TRIF) signaling pathways (10). Further viral RNA is recognized by MDA-5 or RIG-1 in the cytosol, however MDA-5 is much involved in the process of RV (11). Recognition of viruses by PRRs induces the translocation of nuclear factor kappa-light-chain-enhancer of activated B-cells (NF-κB) and interferon regulatory factors (IRF) to the nucleus (10), which release various proinflammatory cytokines and chemokines such as IL-6, IL-8, CCL5, granulocyte macrophage colony-stimulating factor (GM-CSF), and interferons (IFNs) including IFN-λ (10, 12–14) (**Table 1**). However, RV infection inhibits antiviral responses. For example, it activates transforming growth factor (TGF) β, which can increase viral replication (15, 16).

**Table 1:** Characteristics of virus, important viral protein, entry molecules, recognition molecules and downstream cytokines/chemokines of RV, RSV and SAR-CoV-2.

Virus	Type	Important viral protein for infection	Entry molecules (Receptor)	Recognition molecules (PRRs)	Downstream cytokines/chemokines
RV	Positive- sense ssRNA	VP1 VP3	1) Major RV-A and all RV-B ICAM-1 2) Minor RV-A Low-density lipoprotein receptor 3) RV-C CDHR3	TLR3 (dsRNA) TLR7 (ssRNA) MDA-5 RIG-1	1) Proinflammatory cytokines/chemokines IL-1β, IL-6, IL-8, IL-12, TNF-α, CCL5, CXCL9, CXCL10, GM-CSF, IL-33, TSLP, IL-25 2) IFNs IFN-α, IFN-β, IFN-λ
RSV	Negative-sense ssRNA	G protein F protein SH protein	1) G protein Glycosaminoglycans CX3CR1 Annexin II 2) F protein Nucleolin, Co-receptor EGFR, TLR4 and ICAM-1	TLR3 (dsRNA) TLR7 (ssRNA) TLR4 (F protein) RIG-1 MDA-5	
SARS-CoV-2	Positive- sense ssRNA	S1 S2	ACE2 TMPRSS2 (for proteolytic cleavage)	TLR3 (dsRNA) TLR7 (ssRNA) MDA-5 RIG-1	

Respiratory syncytial virus (RSV) is known to cause bronchiolitis with wheezing in infants. In adults, it can cause community-acquired pneumonia, and mortality rates comparable to influenza have been reported in elderly and high-risk patients (17). RSV is also known to be involved in the exacerbation of asthma. RSV is more frequently detected than RV in patients with wheezing less than 3 years of age. Although there is little data for adults, it has been reported that RSV is involved in 7% of asthma hospitalizations (18). RSV is negative-sense ssRNA virus and includes 10 proteins including 3 surface proteins such as fusion (F) protein, attachment (G) protein and small hydrophobic (SH) protein (**Table 1**). G protein is responsible for viral attachment and, glycosaminoglycans, CX3CR1, and annexin II are proposed for cell receptors of RSV G protein (19, 20) (**Table 1**). F protein is critical for cell fusion, resulting in viral entry and infection. A cellular receptor for RSV F protein is nucleolin (21) and TLR4, EGFR, and ICAM-1 are reported to be co-receptor (19, 20) (**Table 1**). Fulin as a protease play a role in the intracellular cleavage of F protein, which is essential step for acquirement of RSV infectivity. RSV replicates in airway epithelial cells, and TLR3 and TLR7/8 in the endosome of epithelial cells play roles in the recognition of dsRNA and ssRNA in a similar way of RV. RSV RNA is also recognized by RIG-1 or MDA-5 in the cytosol. Further, F protein is recognized by TLR4 (22) (**Table 1**) expressed in the cell. Proinflammatory cytokine/chemokines and IFNs are then induced.

Clinically, it is not always symptomatic even if virus is detected, As a result of this, it is important to clarify the factors that determine the severity of viral infection. Candidates are (1) host-related factors, (2) virus-related factors, and (3) gene-related factors. As for host-related factors, several reports suggest that asthmatic patients are more susceptible to virus including RV, and their symptoms are easily exacerbated by RV infections (23, 24). As a potential mechanism, antiviral cytokines such as IFNs are produced at lower levels in asthmatic patients compared to non-asthmatic patients (25, 26), which is discussed later.

Virus-related factors have recently been highlighted. Several reports have noted differences in virulence between RV species; specifically, RV-C cause more serious pathogenic diseases than other RVs, including RV-B (13, 27–29). Furthermore, the reason why RV is much involved in the pathogenesis of asthma exacerbation has not been fully clarified. RSV infection is related to the induction of Th2-stimulated immune responses (30). CX3CR1, a receptor for RSV G protein, and its ligand CX3CL1 exacerbates allergic immune responses (31). However, if compared to the case of RV infection, IL-5 concentration in the serum during RSV infection is not increased (32), suggesting that the degree of type-2 bias may be lower than that in RV infection. There is a possibility that stronger viral responses, probably mediated by IFNs, suppress Th2-mediated immune responses and weaker viral responses by RV upregulates Th2 immune responses.

As for gene-related factors, recent studies suggest that interactions between genes and viral infection may play a role in the pathogenesis of asthma exacerbation. For example, a coding single nucleotide polymorphism (SNP) in CDHR3 (rs6967330; C_529_Y) is associated with severe exacerbation in childhood asthma (33). Since then, CDHR3 has been found to be a receptor for RV-C (9). Moreover, this SNP enhances the protein expression of CDHR3 on the cell surface (9, 33), which increases the binding of RV-C and its replication (9). This SNP also increases RV-C illnesses *in vivo* (34), which suggests that asthma is easily exacerbated in patients with CDHR3-Y_529_ variants by increased susceptibility to RV-C.

Since 2020, the novel coronavirus severe acute respiratory syndrome coronavirus 2 (SARS-CoV-2) that cause Coronavirus disease 2019 (COVID-19) has spread globally and created pandemic. SARS-CoV-2 is a positive-sense ssRNA virus and enters host cells by the surface S protein comprising S1 and S2 (35). The receptor for SARS-CoV-2 is angiotensin-converting enzyme 2 (ACE2) (**Table 1**). TMPRSS2 play a role in the intracellular proteolytic cleavage which induces a conformational change in S protein and allows for cellular entry *via* endocytosis. SARS-CoV-2 replicates in airway epithelial cells, and TLR3 and TLR7/8 in the endosome and MDA-5 or RIG-1 in the cytosol play roles in the recognition of viral components in a similar way. Severe cases of COVID-19 are assumed to be due to the defect or delay of IFN responses which unlease excessive expression of proinflammatory cytokines/chemokines, called cytokine storm syndrome (35). Furthermore, other mechanism such as increase in ACE2 signaling, observed in the case of SARS-CoV (36), may contribute to the immune response to SARS-CoV-2. ACE2 is not the only receptor mediating SAR-CoV-2 cell entry. For example, neuropilin-1 and DPP4 is reported to be potential receptor for SAR-CoV-2 (35). Asthma patients are reported to be less likely to suffer from COVID-19 or its severe disease (37). However, whether asthma is associated with severity of COVID19 is still controversial (37, 38). Zhu et al. reported that although the risk of severe COVID-19 is not elevated in patients with allergic asthma, it is significantly increased in those with non-allergic asthma.

In recent years, there have also been reports that mixed infections of viruses and bacteria (e.g., *Moraxella catarrhalis*) are involved in the exacerbation of asthma (39).

## Viral Infection in Airway Epithelial Cells

### 1) General Roles/Responses of Airway Epithelial Cells in Viral Infection

Virus infects airway epithelial cells. Generally, virus is taken up by receptor-mediated endocytosis and replicates in epithelial cells as described above. The components of the viruses are recognized by PRRs including TLRs, MDA-5, and RIG-1. dsRNA is recognized by TLR3, and ssRNA is recognized by TLR7/8 in endosomes, and viral RNA is recognized by RIG-1 or MDA-5 in the cytosol. RSV F protein is recognized by TLR4 in the cell surface (22). Recognition of viruses by PRRs induces the translocation of IRF and NF-κB to the nucleus and the transcription of proinflammatory and antiviral genes such as IFNs

### 2) Status of Airway Epithelial Cells in Asthma Patients

When an asthmatic patient is infected with RV, the symptoms of the upper respiratory tract are similar to those of non-asthmatic subjects (23, 24). However, the symptoms of the lower respiratory tract such as coughing are exacerbated in asthmatic patients (23, 24). In addition, RV is frequently detected in the lower airways of asthmatic patients even during the stable period (40). As a potential mechanism, antiviral cytokines such as IFN-β and IFN-λ are produced at lower levels in asthmatic patients than in non-asthmatic patients (25, 26) (**Figure 2**), and thus viral replication is higher in asthmatic patients. However, the hypothesized lower production of antiviral cytokines from airway epithelial cells and higher viral replication in asthmatics is still controversial, as several reports suggest that these factors are almost the same as in non-asthmatics (41–43). In contrast, the expression of PPRs such as TLR3, MDA-5 or RIG-1 in epithelial cells of asthma is similar to that of healthy volunteer (44).

As for the receptor of virus, the expression of ICAM-1 is upregulates in asthma (45) and its expression further increases after RV infection (46), which can induce the eosinophilic airway inflammation as described below. In contrast, expression of ACE2 is decreased by IL-4 or IL-13 (47) and its expression is decreased by allergen challenge (48), suggesting that the expression of ACE2 is lower in asthmatic patient than in healthy indivisuals.

## Involvement of Dendritic Cells During Viral Infection

### 1) General Roles/Responses of Dendritic Cells (DCs) in Viral Infection

DCs play important roles in innate immune responses during viral infection (10). DCs are professional antigen presenting cells, and present antigens such as viral components to naïve T cells. DCs are classified into myeloid DCs (mDCs) and plasmacytoid DCs (pDCs). mDCs are involved in the initiation of the cytotoxic T-cell response and the activation of T helper cells. mDCs differentiate naïve T cells into effector T cells. Th1 cells contribute to the anti-viral responses, and Th2 cells contribute to allergic inflammation. mDCs express TLR4, and stimulation by TLR4 upregulates DC functions. RSV F protein can activate mDC functions through TLR4.

**Figure 1:**
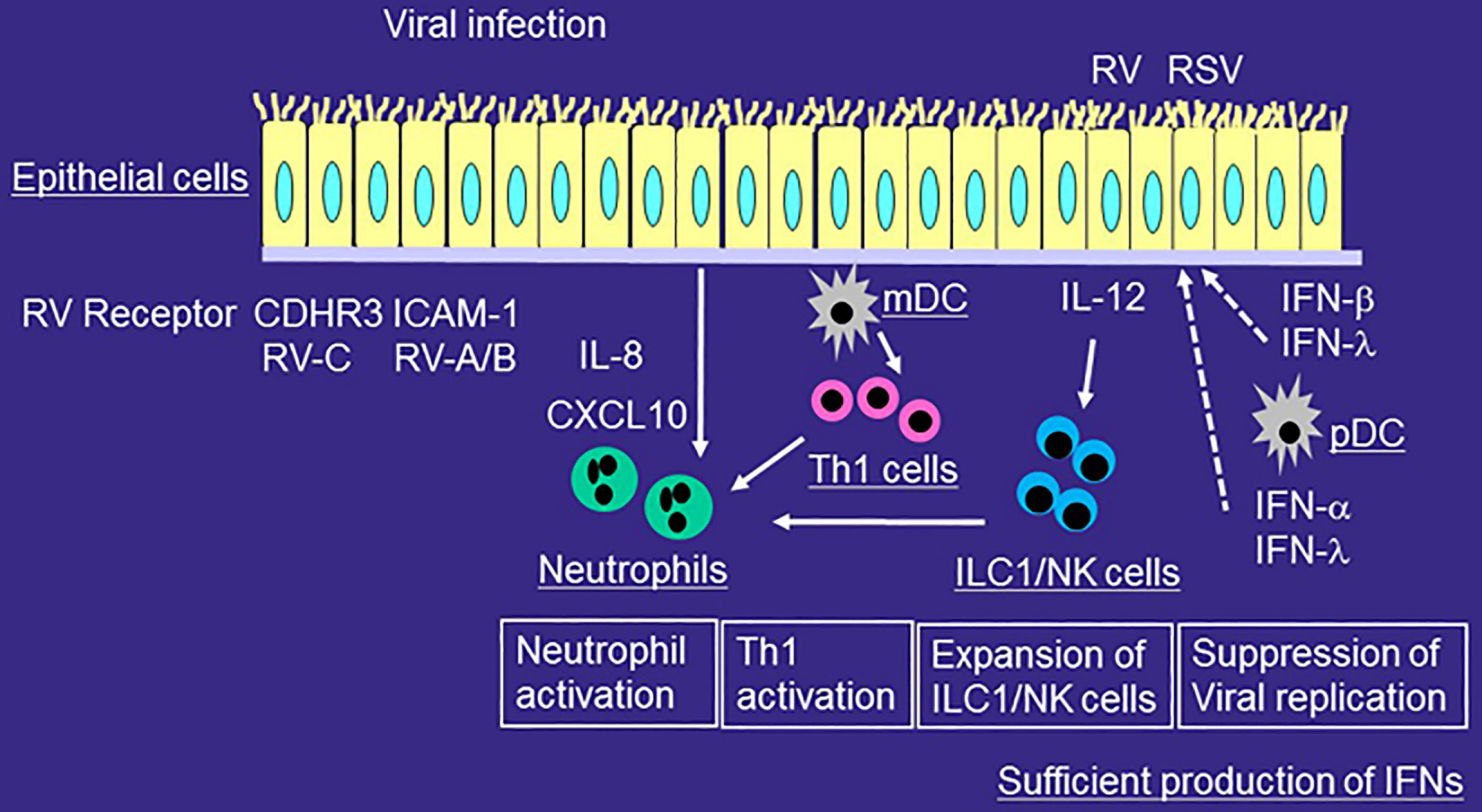
Role of innate immune responses in the development of airway inflammation of healthy individuals during viral infection. RV or RSV infects airway epithelial cells and is taken up by receptor-mediated endocytosis. After recognition of virus components by PRRs, epithelial cells release proinflammatory cytokines and chemokines and antiviral cytokines including IFNs. mDCs present viral antigens to naïve T cells for differentiation, whereas pDCs produce IFN-α and IFN-λ. The production of IFN from airway epithelial cells or pDCs is sufficient for viral immunity. RV or RSV infection induces more IL-12 expression in airway epithelial cells, and more ILC1 and NK cells in the airways. Viral infection releases IL-8 from epithelial cells and thus induces neutrophilic airway inflammation. Airway neutrophils disappears relatively quickly and eosinophilic airway inflammation is not usually induced.

In contrast, pDCs contribute to the induction of tolerance and the maintenance of homeostasis in the lungs. pDCs produce large amounts of IFN such as IFN-α and IFN-λ through TLR7/8 for anti-viral immune responses (**Figure 1**) and induce regulatory T cells. Important role of pDC in the development of severe COVID-19 is proposed. SAR-CoV-2 can avoid or delay the stimulation of type I IFN-related responses *in vivo*. pDC tune down their capacity for IFN production, which can favor prolonged viral replication, termed “pDC exhaustion”

### 2) Status of DCs in Asthma Patients

mDCs of airway in asthmatic patients are increased and activated as compared with those in healthy individuals (49), which contribute to the exacerbated allergic immune responses in asthma. There is a possibility that it is also involved in the exacerbated viral-related immune responses.

**Figure 2:**
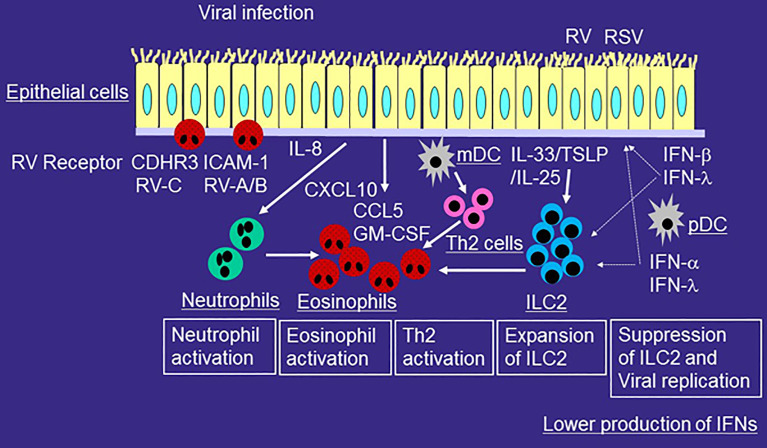
Role of innate immune responses in the development of type 2-mediated airway inflammation of asthmatic patients during viral-induced asthma exacerbation. RV or RSV infects airway epithelial cells and is taken up by receptor-mediated endocytosis. After recognition of virus components by PRRs, epithelial cells release proinflammatory cytokines and chemokines including IL-6, IL-8, CCL5, GM-CSF, and CXCL10 and antiviral cytokines such as IFN-β and IFN-λ. mDCs present viral antigens to naïve T cells for differentiation, whereas pDCs produce IFN-α and IFN-λ. The production of IFN from airway epithelial cells or pDCs is lower in patients with asthma or with high IgE as compared with that of healthy individuals. RV or RSV infection induces more IL-33, IL-25, or TSLP expression in airway epithelial cells. Virus-induced IL-33, IL-25, or TSLP increase ILC2s in asthmatic airways and thus induce eosinophilic airway inflammation. IL-33 enhances RV-induced airway inflammation and suppresses IFN-β or IFN-λ expression and anti-viral immunity. Viral infection releases IL-8 from epithelial cells and thus induces neutrophilic airway inflammation. Activated neutrophils can accumulate eosinophils in the airway even without chemoattractants for eosinophils. Viral infection releases a variety of mediators including CCL5, GM-CSF, and CXCL10, which can directly activate eosinophils. Receptors of RV such as ICAM-1 and CDHR3 in airway epithelial cells also activate eosinophils.

IFN production from pDCs decreases in patients with high IgE levels or in those with asthma (50, 51) (**Figure 2**). For example, the production of the anti-viral cytokine IFN-α from influenza-stimulated pDCs is inversely correlated with the concentration of serum IgE (50). IgE cross-linking of peripheral blood mononuclear cells (PBMCs) suppresses the production of RV-stimulated anti-viral cytokines IFN-α and IFN-λ in asthmatic patients (51).

### 3) Discussion About the Status of DCs in Asthma Patients

Reflecting the reduced IFN production from pDCs, asthma is easily exacerbated by RV infection in patients with high IgE levels. Experimental infection with RV increases AHR and fractional exhaled nitric oxide (FeNO) in patients with high serum IgE (52). Asthma exacerbation is more likely to occur in patients with high concentrations of specific IgE during RV infection (53). Furthermore, anti-IgE Ab treatment reduces pDC surface receptor (FcϵRIα) expression and restores RV-induced IFN-α production from pDC (54). Anti-IgE Ab decreases the duration of RV infection, peak RV shedding and the frequency of RV illnesses (55), Moreover, anti-IgE Ab reduces acute severity of RV-induced asthma exacerbation (56). Therefore, IgE plays an important role in the IFN production from pDC and RV-induced asthma exacerbation

## Interactions of Viral Infection and Neutrophils or Eosinophils in the Development of Asthma Exacerbation

### 1) General Roles/Responses of Neutrophils or Eosinophils in Viral Infection

Viral infection induces neutrophilic airway inflammation mainly due to the release of cytokine/chemokines from epithelial cell. Neutrophils play roles in enhancing viral-induced inflammation through releasing granules, producing cytokines, and inducing the recruitment of other immune cells in the airways (**Figure 1**). IL-8 contributes to the accumulation of neutrophils in the site of inflammation, and IL-8 expression is upregulated in the airways of patients with viral infection (57, 58). However, in healthy individuals, airway neutrophils disappears relatively quickly (59). Eosinophilic airway inflammation is not usually induced (59).

### 2) Status of Neutrophils or Eosinophils in Asthma Patients

Both neutrophil and eosinophil inflammation may be involved in the pathogenesis of severe asthma. Viral infection induces neutrophilic airway inflammation as described above, which contributes to the exacerbations that frequently occur in severe asthma. The European Network For Understanding Mechanisms Of Severe Asthma (ENFUMOSA) reported that higher sputum neutrophil counts and mediators derived from eosinophils are observed in patients with severe asthma (60). In fact, IL-8 expression is upregulated in the airways of patients with severe asthma (61, 62).

The number of eosinophils also increases in the asthmatic respiratory tract during or after viral infection. Experimental infection with RV increases the accumulation of eosinophils in the airways after allergen challenge in patients with allergic rhinitis (63). Viral infection increases the eosinophil number in airway epithelium (59) and the levels of eosinophil cationic proteins in sputum (64) in patients with allergic asthma. This shows that eosinophils are actually activated and recruited in the asthmatic respiratory tract during or after viral infection (**Figure 2**).

### 3) Discussion About the Status of Neutrophils or Eosinophils in Asthma Patients

Recent studies have suggested that activated neutrophils can induce eosinophilic airway inflammation. For example, neutrophil extracellular traps (NETs) play a role in the development of eosinophilic airway inflammation of viral-induced asthma exacerbation. RV infection causes the release of dsDNA with the formation of NETs in a mouse model (65). In addition, in humans, the release of host dsDNA after RV infection is correlated with the exacerbation of type 2 allergic inflammation (65). Furthermore, neutrophil proteases, including elastase, directly activate eosinophil functions such as the production of superoxide anions and cytokines, and eosinophil cationic protein release (66, 67). Moreover, we reported that IL-8 or LPS-stimulated neutrophils induce the trans-basement membrane migration of eosinophils *in vitro* even without eosinophil chemoattractants (68, 69). Therefore, activated neutrophils can accumulate eosinophils in the asthmatic airways during viral-induced asthma exacerbation (70) (**Figure 2**).

To accumulate in asthmatic airways, circulating eosinophils need to adhere to vascular endothelial cells, migrate over cells, and be activated locally by inflammation (71). In this process, adhesion molecules such as vascular cell adhesion molecule (VCAM) 1 or ICAM-1, chemokines such as CCR3 ligands including CCL11 (eotaxin-1), and cytokines such as IL-5 play important roles. RV infection induces CCL5 (13, 14), a CCR3 ligand, and GM-CSF (12), an eosinophil growth factor/cytokine, which contribute to the migration and activation of eosinophils in the airway during viral infection.

Chemokines other than CCR3 ligands also play roles in the pathogenesis of RV-induced asthma exacerbation. For example, RV infection induces CXCL10, a CXCR3 ligand, in airway epithelial cells *in vitro* and *in vivo* (13, 72). Specifically, serum CXCL10 levels increase in virus-induced asthma and correlate with the severity of the disease, including airflow limitations (72). CXCL10 directly upregulates eosinophil functions *via* CXCR3 expressed in eosinophils (73).

As describe above, ICAM-1 is an adhesion molecule (74); however, it is also the cell receptor of RV-A and RV-B (8). ICAM-1 expression in epithelial cells increases after RV infection (46), and ICAM-1 directly upregulates eosinophil functions (75, 76). CDHR3 is a receptor for RV-C (9), and its SNPs are related to severe exacerbations (33). We recently reported that CDHR3 activates eosinophil functions (77). These findings suggest that ICAM-1- and CDHR3-mediated adhesion of eosinophils to epithelial cells may activate eosinophils during RV-induced asthma exacerbation (**Figure 2**). In addition, transfection of CDHR3-Y_529_ into HeLa cells increases eosinophil adhesion and superoxide anion production compared to CDHR3-C_529_ or negative controls, suggesting a possible role for CDHR3-induced eosinophil activation in the development of asthma exacerbation, especially with the CDHR3 variant.

Eosinophils are known to have an antiviral effect (78, 79). For example, eosinophils directly suppress the replication of parainfluenza virus through NO production (79). In addition, eosinophils directly suppress RSV replication through eosinophil-derived neurotoxin release (78). However whether the antiviral effect of eosinophils is actually exerted in the asthmatic airways is still unknown.

On the other hands, eosinophilic inflammation can also be a risk factor for RV-induced asthma exacerbations through the suppression of IFN production (80–84). Both high FeNO and sputum eosinophilia increase the risk of subsequent virus-induced asthma exacerbation (82). There are no reports that eosinophils directly suppress RV replication. Mathur et al. reported that eosinophils from allergic rhinitis can suppress the RV-induced IFN-λ1 expression from epithelial cells (BEAS-2B cells) *in vitro* and thus increase RV replication, probably through TGF- β (80). Recently, Dill-McFarland et al. reported that eosinophils or eosinophil supernatants inhibites RV-induced IFNα secretion from pDC of healthy volunteers *in vitro* (84). Furthermore, anti-IL-5 treatment increases (or restores) RV-induced IFNα secretion from pDC of asthmatic donor *ex vivo* as compared with that without anti-IL-5 treatment (84). Moreover, patients with eosinophilic inflammation demonstrate reduced TLR7 and IFN-λ expression in bronchial epithelial cells (81). Given these, IFN production from epithelial cells or pDC can be inhibited by eosinophils, and reducing eosinophil counts may be an important strategy for controlling RV-induced asthma exacerbations.

As for COVID-19, pre-existing eosinophilia is protective from COVID-19-associated admission in asthmatics, and development of eosinophilia during hospitalization is associated with decreased mortality (85), suggesting the protective role of eosinophils in SARS-CoV-2. However, the direct suppressive effect of eosinophils on SARS-CoV-2 replication has not been demonstrated until now. On the other hands, IFN-α or IFN-γ induces the expression of ACE2 in airway epithelial cells (86) and expression of IFNs is lower in patients with high eosinophil counts (87), suggesting that eosinophil-mediated suppression of IFN production in epithelial cells may be associated with suppression of ACE2 expression and thus COVID-19.

## Role of ILCs in the Pathogenesis of Viral-Induced Asthma Exacerbation

### 1) General Roles/Responses of ILCs in Viral Infection

ILCs play important roles in the viral-induced innate immune responses. ILCs do not express T cell receptor and thus cannot response specific antigens unlike T cells. ILCs are functionally classified based on expression patterns of transcription factor and cytokines; ILC1, which express the transcription factor T-bet and produce IFN-γ, ILC2, which expresses the transcription factor GATA3 and produce IL-5 and IL-13, and ILC3, which expresses the transcription factor RORγt and produce IL-17A or IL-22. In healthy individuals, ILC1 and NK cells, another effector lymphocytes of the innate immune system, are involved in the immune responses of viral infection as well as bacterial infection through the production of IFN-γ (**Figure 1**).

### 2) Status of ILCs in Asthma Patients

Innate immune responses are also involved in the pathogenesis of eosinophilic airway inflammation; this process includes ILC2 as well as epithelial cell-related cytokines such as IL-33, TSLP, and IL-25 (6, 7). The ILC2 activated by IL-33, TSLP, and IL-25 can produce IL-5 and IL-13 and thus induce eosinophilic inflammation. Several reports suggest that ILC2 is increased in the blood or airways of patients with asthma (88, 89) and highly increased in severe asthma as compared with mild asthma (90, 91). For example, ILC2 is increased in the blood and sputum of patients with severe asthma (91). However, in blood, the finding of increased amounts or frequency of ILC2 in asthma is controversial (89, 92), as the frequency of blood ILC2 has been found not to differ between well-controlled asthma and uncontrolled asthma (89). Rather, ILC2 in severe asthma is more activated than in mild asthma (89, 91). For example, IL-5^+^ ILC2 in peripheral blood and sputum of severe asthma patients is increased as compared to those with mild asthma or control patients (91). Furthermore, IL-13^+^ ILC2 is increased in the peripheral blood of patients with uncontrolled asthma (89). As for the mechanism of ILC2 induction, allergen exposure increases ILC2 in the airways and decreases them in blood (93, 94), suggesting the accumulation of ILC2 from circulation into airways in response to allergens. Recently, the role of TSLP in the pathogenesis of severe asthma has been highlighted. TSLP contributes to the pathogenesis of corticosteroid-resistant airway inflammation by Bcl-xL expression *via* ILC2s (95, 96). From the above, it can be seen that ILC2 contributes to the development of airway inflammation in severe asthma.

Recent studies have demonstrated that innate immune responses including ILC2 play important roles in virus-induced asthma exacerbation. For example, Jackson et al. reported that RV-16 inoculation induces not only IL-4, IL-5, and IL-13 but also IL-33 in the asthmatic airway *in vivo*, and these are related to the severity of exacerbation; furthermore, IL-33 induction correlates with viral load and the induction of IL-5 and IL-13 (97). Dhariwal et al. examined the ratio of pulmonary ILC2 and ILC1 in asthma after RV-16 inoculation as compared with that of non-asthmatic subjects (98). They found that the ratio of ILC2 to ILC1 in bronchoalveolar lavage cells of asthmatics at baseline and after RV-16 inoculation is higher than that of non-asthmatic subjects, and it correlates with the severity of exacerbation and the induction of type 2 cytokines in nasal fluid (98). These findings suggest that ILC2 contributes to the development of type 2-mediated airway inflammation in viral-induced asthma exacerbation (**Figure 2**).

### 3) Discussion About the Status of ILCs in Asthma Patients

Epithelial cell-related cytokines such as IL-33, IL-25, and TSLP are induced by viral infection. RV produces IL-33 from airway epithelial cells or from bronchial smooth muscle cells *in vitro* (97, 99, 100), and supernatants of RV-infected bronchial epithelial cells induce type 2 cytokines from human T cells and ILC2 (97). RV induces IL-25 from airway epithelial cells of asthmatic patients *in vitro* (101). RSV induces TSLP in airway epithelial cells *in vitro via* activation of the innate signaling pathway (102). These findings suggest that viruses directly induce or produce epithelial cell-related cytokines, which can contribute to the induction of ILC2 and eosinophilic inflammation. However, the production of epithelial cell-related cytokines may depend on the type of virus, type of cells, the presence or absence of asthma, time of infection, and experimental conditions. Given the above, the actual role of epithelial cell-related cytokines in the pathogenesis of viral-induced asthma exacerbation needs to be further clarified. For example, the production of IL-33 by RV is much lower than that of other cytokines/chemokines such as IL-6 or IL-8 (100). We infected sinus or bronchial epithelial cells that were differentiated at the air-liquid interface with RVs, including RV-C, and measured the concentrations of IL-33, TSLP, and IL-25 in the basal medium; however, these factors were not induced (data not shown), in contrast to CCL5, CXCL10, CXCL11, IL-6, and IL-8 (13).

Mouse models are important for investigating host immunity during virus infection. However, mouse models of RV-infection have not always reflected human infection, because major groups of RVs such as RV-16 do not bind to mouse ICAM-1 and thus do not infect mice (103). As a result, minor groups of RVs such as RV-1B are often used in mouse models of RV infection. Using RV1B and RV-infected immature mice, Han et al. examined the roles of IL-33 and TSLP in RV-induced airway inflammation and ILC2 expansion; RV1B infection increased the expression of IL-33 and TSLP in the airway (104). RV1B-induced mucous metaplasia, expansion of ILC2, and AHR were suppressed by treatment with anti-IL-33 Ab or deletion of the TSLP gene (104). Beale et al. reported that RV1B infection increases pulmonary IL-25 expression, which is associated with increased type 2 cytokine production and increased viral load (101). Blockade of the IL-25 receptor reduces many RV-induced exacerbation-specific responses, including type 2 cytokine expression (101). Furthermore, Hong et al. reported that RV1B infection induces lung IL-13 and IL-25, and IL-13-producing ILC2 in neonatal mice, while an anti-IL-25 Ab suppresses ILC2 expansion, mucus hypersecretion, and AHR (105). As for RSV, Stier et al. reported that RSV infection upregulates IL-13-producing ILC2 with IL-13 expression in the lung (106). They also found that anti-TSLP Ab treatment or TSLP receptor deletion suppresses IL-13-producing ILC2 (106). These findings indicate important roles for IL-33, IL-25, and TSLP in the development of RV or RSV-induced ILC2-mediated airway inflammation in mice.

Recently, Rajput et al. developed a mouse model of RV-C infection; using immunofluorescence, they verified the colocalization of RV-C15 and CDHR3 in mouse ciliated airway epithelial cells (107). They reported that RV-C15-infected mice demonstrate greater eosinophilic airway inflammation; expression of IL-5, IL-13, IL-25, IL-33, and TSLP; and expansion of ILC2 compared to RV-A1B-infected mice (107). It was also found that RV-C-infected *Rora^fl/fl^ Il7r^cre^* mice deficient in ILC2 do not develop eosinophilic inflammation or the expression of IL-13 mRNA (107), suggesting that RV-C infection induces ILC2-mediated type 2 airway inflammation in mice. Different patterns of RV-induced airway inflammation among RV species are of great interest and should also be examined in humans.

Recent studies suggest that IL-33 exacerbates RV-induced airway inflammation and reduces anti-viral immunity. IL-33 increases the RV-16-induced inflammatory activity of human lung vascular endothelium and viral replication *in vitro* (108). IL-33 increases RV-induced type 2 cytokine production from PBMCs of asthmatics, but not of non-asthmatics (109). Werder et al. reported that an anti-IL-33 Ab decreases airway inflammation of cockroach-sensitized and challenged RV-infected mice (110). It also decreases RV replication and increases IFN-λ expression in mouse lungs *in vivo* and in human airway epithelial cells *in vitro* (110). In addition, Ravanetti et al. reported that IL-33 increases asthmatic airway inflammation and AHR in house dust mite-sensitized and challenged influenza-infected mice (111). They also show that an anti-ST2 Ab, which antagonizes IL-33, increases the expression of IFN-β in epithelial cells and DCs (111). As such, especially in RV infection, IL-33 plays roles in the augmentation of viral-induced asthma exacerbation.

## Possible Treatment Strategies for Suppressing Viral Infection and ILC2 Activation

Type I IFNs including IFN-α and IFN-β, type II IFNs including IFN-γ, and type III IFNs including IFN-λ have anti-viral capacity *in vitro* (112–115). IFN-β and IFN-γ also suppress the activation of ILC2 *in vitro* (116). This means IFN-β or IFN-γ can suppress not only viral replication but also ILC2 activation *in vitro*, which could be a novel strategy for treating viral-induced asthma exacerbation.

Low-dose IFN-α treatment improves lung function and allows for decreased corticosteroid dose in severe asthma (117). In a mouse model of allergic airway inflammation, IFN-γ attenuates RV1B-induced IL-13 expression and mucous metaplasia in immature mice, with a reduction in the expansion of ILC2s and the expressions of IL-5, IL-13, IL-17RB, ST2, and GATA-3 mRNAs in ILC2s (118). IFN-γ treatment also suppresses the allergen-induced overall immune response in a mouse model (119).

As systemic administration of IFN-α, IFN-β, or IFN-γ increases the risk of developing auto-immune diseases such as systemic lupus erythematosus (120) and liver dysfunction, the administration of IFN by inhalation has been developed. For example, Djukanović et al. examined the effect of inhaled IFN-β on cold-induced asthma exacerbation. Although IFN-β had no clinical benefit in treating asthma, it improved Asthma Control Questionnaire-6 responses in severe asthma patients in an exploratory analysis (121). Inhaled IFN-β tend to suppress RV load in sputum, whereas it boosted innate immunity as assessed by blood and sputum anti-viral biomarkers such as OAS1 and Mx1 (121). The effect of inhaled IFN-β on COVID19 has also been investigated (122), and available data from a phase II study demonstrate that inhaled IFN-β can accelerate recovery from the disease.

Another novel approach to suppress viral infection and ILC2 expansion is TLR agonists. For example, TLR7 agonists induce IFNs (123), which have anti-viral properties *in vitro*. TLR7 activation increases IFN-λ receptor mRNA expression in PBMCs (124). Furthermore, a TLR7 agonist has been reported to inhibit ILC2-dependent airway inflammation through interstitial macrophages producing IL-27 in a mouse model (125). Another potential approach is TLR3 agonism. TLR3 agonists induce IFN-β, which antagonizes STAT5-activating cytokines and suppresses ILC2 responses in lungs in a mouse model (126). These findings suggests that TLR agonism could be an important strategy for the suppression of viral-induced ILC2 activation, and thus viral-induced asthma exacerbation.

Anti-TSLP Ab treatment reduces asthma exacerbation in severe asthmatics (127) and clinical study demonstrates that anti-TSLP Ab decreases IL-5 and IL-13 concentrations in serum and eosinophilic inflammation in the airway (128). Although anti-TSLP Ab is assumed to suppress viral-induced ILC2-medicated eosinophilic inflammation as demonstrated in mouse model (106), actual effect of anti-TSLP Ab on viral-induced innate responses and ILC2 activation in patients with asthma should have been examined.

## Conclusion

ILC2 plays important roles in the development of type 2-mediated airway inflammation in viral-induced asthma exacerbation. IFNs including IFN-β or TLR agonists can suppress not only viral replication but also ILC2 activation, which could become an innovative strategy for the treatment of virus-induced asthma exacerbation.

## Author Contributions

KN wrote the manuscript. MN edited the manuscript. All authors read and approved the final manuscript.

## Funding

This work was supported by a grant from the Ministry of Education, Culture, Sports, Science, and Technology (15K09228).

## Conflict of Interest

The authors declare that the research was conducted in the absence of any commercial or financial relationships that could be construed as a potential conflict of interest.

## Publisher’s Note

All claims expressed in this article are solely those of the authors and do not necessarily represent those of their affiliated organizations, or those of the publisher, the editors and the reviewers. Any product that may be evaluated in this article, or claim that may be made by its manufacturer, is not guaranteed or endorsed by the publisher.
